# Weiterbildung in der Praxis!

**DOI:** 10.1007/s00117-025-01517-y

**Published:** 2025-09-22

**Authors:** Mina Soltani, Isabel Molwitz, Mike Notohamoprodjo

**Affiliations:** 1 Klinik und Poliklinik für Diagnostische und Interventionelle Radiologie und Nuklearmedizin, Zentrum für Radiologie und Endoskopie, Universitätsklinikum Hamburg-Eppendorf, Hamburg, Deutschland; 2 Radiologische, Strahlentherapeutische und Nuklearmedizinische PartG, München, Deutschland

Das Fachgebiet der Radiologie bietet die besondere Möglichkeit, die Weiterbildung sowohl in Kliniken (einschließlich Universitätskliniken und Lehrkrankenhäusern) als auch in niedergelassenen Einrichtungen zu absolvieren [1]. Diese duale Option eröffnet ein breites Spektrum an Ausbildungswegen und erlaubt es angehenden Fachärzt:innen, unterschiedliche Arbeitsumfelder kennenzulernen. Dennoch sind Weiterzubildende in der Niederlassung die Minderheit.

Dieser Artikel bietet einen Überblick über die Vor- und Nachteile der jeweiligen Ausbildungsstätten. Die Wahl des Weiterbildungsorts kann den Karriereverlauf und eine Spezialisierung beeinflussen. Daher sollte die Entscheidung zur Wahl der Ausbildungsstätte informiert und wohlüberlegt erfolgen.

## Weiterbildungsdynamik und strukturierte Weiterbildung

Der Einstieg in die radiologische Weiterbildung kann in einer Praxis anders strukturiert sein als in einer Klinik. Während in Kliniken häufig von Beginn an ein hohes Tempo gefordert wird, um frühzeitig diagnostische Sicherheit für den Dienstbetrieb zu erlangen, gestaltet sich die Einarbeitung in der Praxis je nach Ausrichtung und Arbeitsweise unterschiedlich. Insbesondere kann für Berufsanfänger:innen, die frisch nach dem Studium in die Radiologie einsteigen, der Beginn der radiologischen Befundung in einer Praxis unterschiedlich verlaufen. In vielen Praxen steht zunächst die konventionelle Röntgenbildgebung im Vordergrund, bevor Schnittbildverfahren wie die Computertomographie (CT) und die Magnetresonanztomographie (MRT) hinzukommen. Es gibt jedoch auch Niederlassungen, in denen die MRT-Diagnostik von Anfang an eine zentrale Rolle spielt. Je nach Praxisstruktur und fachlichem Schwerpunkt kann die Ausbildung daher variieren. Mit der Zeit steigt die Anzahl der täglich zu befundenen Untersuchungen in der Praxis jedoch in jedem Fall deutlich an und kann die Untersuchungsanzahl in Kliniken übertreffen. Zudem kann eigenständiges Arbeiten ohne direkte Supervision früher erforderlich sein.

Eine strukturierte Weiterbildung ist in Universitätskliniken weiter verbreitet als in der Niederlassung [2]. So stehen den Assistenzärzt:innen zahlreiche interdisziplinäre Besprechungen und oftmals auch über die Klinik abonnierte digitale Lernangebote zur Verfügung, die eine kontinuierliche Wissensvermittlung und Weiterentwicklung ermöglichen. In der Praxis hingegen sind interne Fortbildungsangebote seltener, weshalb das eigenständige, proaktive Lernen, beispielsweise über Fachliteratur oder die Teilnahme an externen Fortbildungen, eine größere Rolle spielt. Ausnahmen bilden kostenfrei verfügbare Angebote wie RADUCATION – die digitale Lernplattform des Forums Junge Radiologie zur Vorbereitung auf die ersten eigenen Dienste und die Facharztprüfung (www.raducation.de) [3]. Gleichzeitig entfällt in der Praxis teilweise die durch Weiterzubildende als effektivstes Lerninstrument gewertete persönliche Supervision der Befunde [2], da die Befundfreigabe von Befunden durch Fachärzt:innen nicht selten unter hohem Zeitdruck erfolgt, weil die Befunde häufig direkt mit den Patienten besprochen werden. Auch in Kliniken ist eine persönliche Befundsupervision durch Fach- und Oberärzt:innen jedoch nicht an allen Standorten gegeben. Diesbezüglich empfiehlt sich, sowohl für die Praxis als auch die Klinik, eine Hospitation vor Aufnahme einer Tätigkeit, um über Weiterbildungsangebote vor Ort informiert zu sein. Ausschließlich in Universitätskliniken und Lehrkrankenhäusern ist überdies die Möglichkeit zur studentischen Lehre gegeben, die anteilig bereits durch Weiterzubildende durchgeführt wird. Diesbezüglich von Relevanz ist, dass die Lehrtätigkeit in Universitätskliniken keinen negativen Einfluss auf Befundungszeiten oder die Arbeitseffizienz zu haben scheint [4].

## Diagnostisches Spektrum und Spezialisierung

Das diagnostische Spektrum in der radiologischen Praxis umfasst eine breite Palette radiologischer Untersuchungen, insbesondere auch der muskuloskeletalen Radiologie (MSK) sowie neuroradiologische Untersuchungen. Durch diese Vielfalt erwerben Assistenzärzt:innen während ihrer Weiterbildung umfassende Kenntnisse in den verschiedenen Teilbereichen der Radiologie. Zudem profitieren sie in der Niederlassung von direktem Patientenkontakt sowie einem unmittelbaren Austausch mit den Zuweisenden.

In großen Kliniken wird sowohl allgemeine Radiologie praktiziert als auch mit spezialisierten und seltenen Krankheitsbildern gearbeitet. Häufig gelangen Patient:innen durch Überweisung aus Praxen oder kleineren Krankenhäusern in die (Universitäts‑)Klinik. Während in der Praxis oder in kleineren Häusern also in der Regel die Primärdiagnosen gestellt und erste Differenzialdiagnosen erarbeitet werden, liegt der Fokus in größeren Kliniken eher auf seltenen Differenzialdiagnosen oder dem Therapieverlauf. Solch spezialisierte Fälle machen die Weiterbildung in einer Universitätsklinik besonders anspruchsvoll und können auch für wissenschaftliche Analysen verwendet werden. Allerdings kann eine strukturelle Trennung durch spezialisierte Abteilungen, etwa für Neuro- oder Kinderradiologie in großen Kliniken, dazu führen, dass Assistenzärzt:innen der Allgemeinradiologie während ihrer Weiterbildung weniger Einblicke in diese Bereiche erhalten, als in der Niederlassung der Fall.

In der Praxis wird im Vergleich zur Klinik verhältnismäßig mehr MRT-Bildgebung durchgeführt, insbesondere im Bereich der muskuloskeletalen Diagnostik. Dadurch erlangen Assistenzärzt:innen in der Praxis während ihrer Weiterbildung umfangreichere Erfahrung in der muskuloskeletalen MRT-Diagnostik. Die Praxis bietet somit eine ideale Grundlage für eine frühzeitige Spezialisierung in der muskuloskeletalen Radiologie. Dasselbe gilt in der Regel für die Senologie.

Hingegen bieten große Kliniken bessere Voraussetzungen, um eine fundierte Weiterbildung in der interventionellen Radiologie zu erhalten. So wird nicht nur eine größere Anzahl, sondern es werden auch hochspezialisierte interventionelle Eingriffe durchgeführt. Dies ermöglicht es Assistenzärzt:innen in der Klinik, ein potenzielles Interesse an einer Spezialisierung in der interventionellen Radiologie frühzeitig zu evaluieren.

## Dienste und interdisziplinärer Austausch

Ein wesentlicher Bestandteil der radiologischen Weiterbildung ist die Teilnahme an Bereitschafts- und Nachtdiensten. Diese sind in radiologischen Praxen in der Regel nicht vorgesehen oder sie werden teleradiologisch durchgeführt. Für diesen Zweck stellt die Praxis einen entsprechend ausgestatteten Arbeitsplatz oder ein mobiles Endgerät für den häuslichen Bereitschaftsdienst zur Verfügung. Obwohl diese Form der Dienste in Umfragen als weniger belastend empfunden wurde als Anwesenheitsdienste und eine höhere Flexibilität bietet, fehlt weitgehend die Interaktion mit den behandelnden Ärzt:innen, was den fachlichen Austausch einschränkt [2]. Diese Interdisziplinarität, die insbesondere in klinischen Diensten durch den engen Austausch mit Kolleg:innen gegeben ist, stellt eine wertvolle Unterstützung für die diagnostische Entscheidungsfindung dar und trägt maßgeblich zur klinischen Einschätzung komplexer Fälle bei. Zudem ermöglicht die direkte klinische Beteiligung einen intensiveren Lernprozess. Auch können gelegentlich technische Schwierigkeiten in der Teleradiologie auftreten, die den Arbeitsablauf beeinträchtigen [2].

## Forschung und Karriereperspektiven

Ein bedeutender Unterschied zwischen Universitätskliniken und anderen Weiterbildungsstätten Aspekt ist die Möglichkeit zur Forschung, die primär an Universitätskliniken gegeben ist. Dies ist insbesondere für Weiterzubildende relevant, die ein Interesse an wissenschaftlicher Arbeit haben oder eine akademische Karriere anstreben. Sollte kein ernsthaftes Interesse an Wissenschaft und den damit oftmals einhergehenden Tätigkeiten außerhalb der eigentlichen Arbeitszeit bestehen, wäre hingegen die Wahl eines nichtuniversitären Maximalversorgers, Grundversorgers oder einer Weiterbildung in der Niederlassung je nach gewünschtem diagnostisch-interventionellem Spektrum die bessere Wahl.

## Arbeitsbedingungen und Work-Life-Balance

Im Vergleich zu Praxen und nichtuniversitären Krankenhäusern erhalten Assistenzärzt:innen in Universitätskliniken seltener einen Arbeitsvertrag für die gesamte Weiterbildungsdauer. Befristete Verträge und die damit verbundene Unsicherheit erschweren die langfristige Planung und können zu einer erhöhten psychosozialen Belastung führen [2]. Zudem ist erwähnenswert, dass Radiolog:innen in Praxen die Vereinbarkeit von Beruf und Familie als deutlich besser einschätzen als ihre Kolleg:innen in Kliniken [5]. Auch ermöglichen Praxen tendenziell flexiblere Arbeitszeiten. Allerdings müssen auch in der Praxis Kernarbeitszeiten abgedeckt werden, die bis in die späten Abendstunden oder am Wochenende ärztliche Präsenz erfordern können.

## Optimierung der Weiterbildung durch Rotationen

Radiologische Weiterbildungsprogramme stehen vor der Herausforderung, sowohl ein breites Grundlagenwissen über sämtliche Modalitäten zu vermitteln als auch den wachsenden Anforderungen spezialisierter Diagnostik gerecht zu werden [6]. Überdies richtet sich die Vergabe der Weiterbildungsbefugnisse nach dem diagnostisch-interventionellen Spektrum vor Ort. Entsprechend ist eine vollständige Weiterbildungsbefugnis insbesondere an kleineren Standorten oder bei getrennten Instituten für Neuro- und Kinderradiologie oftmals nicht gebündelt in der Person eines Weiterbildungsbefugten gegeben. Eine mögliche Lösung, um Weiterzubildenden dennoch eine sichere Perspektive hinsichtlich der Vermittlung sämtlicher Bereiche der Radiologie zu gewähren und inhaltlich suffiziente Expertise bspw. in der MSK-Radiologie oder Senologie aufzubauen, sind Rotationen zwischen ambulanten und klinischen Einrichtungen [7]. Diese sollten verpflichtend gestaltet sein, um zu vermeiden, dass die Teilnahme an einer Rotation in die Niederlassung als fehlendes Interesse an einer klinischen Laufbahn wahrgenommen wird. Rotieren sämtliche Kolleg:innen, reduziert dies auch die Wahrscheinlichkeit eines einseitigen Verlusts von Weiterzubildenden an die Klinik respektive die Niederlassung als potenzielle Sorge von Weiterbildungsbefugten. Entsprechend wird die Sicherstellung einer umfassenden Weiterbildung, beispielsweise durch strukturierte Rotationen, auch durch die Kolleg:innen des Forums Junge Radiologie, der jungen Neuroradiologie und der jungen Kinderradiologie gefordert [8]. Überdies ist damit zu rechnen, dass sich mit Umsetzung des Krankenhausversorgungsverbesserungsgesetzes (KHVVG) und der konsekutiven Fokussierung der Kliniken auf bestimmte Leistungsgruppen das Spektrum der Weiterbildung vor Ort verändern wird und Rotationen notwendiger werden. Hinsichtlich der Länge von Rotationen zeigen Umfragedaten, dass eine mindestens sechsmonatige Rotation bspw. für Interventionen sinnvoll wäre, da kürzere Zeiträume die Ausbildungsqualität und Zufriedenheit der Weiterzubildenden beeinträchtigen [9]. Eine enge Kooperation zwischen Praxen und Kliniken ist auch deshalb hilfreich, da sie nicht nur die Qualität der Ausbildung optimiert, sondern den Weiterzubildenden auch flexiblere Karrierewege ermöglicht. So sollte es auch möglich sein, die Weiterbildung in einer Praxis zu beginnen und im Verlauf an eine Universitätsklinik zu wechseln, mit der Option, zu einem späteren Zeitpunkt in die Forschung einzusteigen. Auch fördern Rotationen den Austausch und das Verständnis zwischen in Krankenhäusern und in der Niederlassung tätigen Kolleg:innen.

## Fazit

- Die radiologische Weiterbildung in Deutschland ist in der Klinik oder Praxis möglich, wobei beide Bereiche spezifische Vor- und Nachteile hinsichtlich der Arbeitsbedingungen und des Ausbildungsspektrums bieten.
- Während Praxen ihren Schwerpunkt häufig auf die MSK-Radiologie und Senologie legen, ermöglichen Kliniken Einblicke in die interventionelle Radiologie.
- Tendenziell bieten Universitätskliniken häufiger Fortbildungs- und digitale Weiterbildungsangebote sowie Forschungsoptionen, während Praxen tendenziell flexiblere Arbeitszeiten ermöglichen.
- Nachtdienste in der Klinik sind anspruchsvoller, bieten jedoch durch interdisziplinäre Zusammenarbeit wertvolle Lernmöglichkeiten und fördern die diagnostische Sicherheit, während teleradiologische Dienste in Praxen den direkten fachlichen Austausch begrenzen.
- Eine strukturierte Rotation zwischen Klinik und Praxis verbessert die Ausbildung, sichert ein breiteres Kompetenzspektrum und stärkt den Austausch von in der Klinik und in der Niederlassung tätigen Kolleg:innen. Eine Übersicht der Vor- und Nachteile der jeweiligen Weiterbildungsstätten ist in Tab. 1 dargestellt.

**Tab. 1:** Tabellarische Übersicht der Vor- und Nachteile einer radiologischen Weiterbildung in Praxis und Klinik

Kriterium	Praxis		Klinik	
	Vorteile	Nachteile	Vorteile	Nachteile
*Einstieg und Lernprozess*	Schrittweiser Einstieg, früheres eigenständiges Arbeiten	Weniger Erfahrung mit akuten und zeitkritischen Fällen, Supervision entfällt häufiger	Strukturierte Weiterbildung, interdisziplinäre Fallbesprechungen, Weiterbildungsangebote	Raschere Bearbeitung einer Vielzahl akuter Fälle, z. B. Schockräume
*Diagnostisches Spektrum*	Direkter Patientenkontakt, direkter Kontakt zum Zuweiser, viel MSK-Radiologie	Weniger seltene und komplexe Fälle	Komplexe und seltene Fälle	Weniger Fokus auf MSK, potenziell Mangel an senologischer Diagnostik
*Interventionen und Spezialisierung*	Frühe Spezialisierung in MSK oder Senologie möglich	Kaum Möglichkeiten zur interventionellen Weiterbildung	Breites Spektrum an Interventionen	Eingeschränkte frühe Spezialisierung in MSK oder Senologie
*Weiterbildungsstruktur*	Flexibles, eigenständiges Lernen	Weniger strukturierte Programme	Strukturiertes Curriculum, Fortbildungen und interdisziplinäre Besprechungen	Weniger Eigenständigkeit
*Forschung und Karriere*	Gute Niederlassungschancen	Kaum Forschung	Starke Forschungsanbindung, akademische Laufbahn möglich	Zusatzbelastung durch Forschung und Lehre
*Nachtdienste und Work-Life-Balance*	Keine oder teleradiologische Nachtdienste, planbare Arbeitszeiten, hohe Zufriedenheit	Weniger interdisziplinärer Austausch	Direkte klinische Interaktion, vielfältige Weiterbildungsmöglichkeiten	Hohe Arbeitsbelastung z. B. durch Nacht- und Wochenenddienste, befristete Verträge, Forschung in der Freizeit

## References

[1] Bundesärztekammer (2020) (Muster‑)Weiterbildungsordnung 2018 in der Fassung vom 12./13.11.2020. Bundesärztekammer, Berlin

[2] Oechtering TH, Panagiotopoulos N, Völker M, Lohwasser S, Ellmann S, Molwitz I, Storz C, Winther H, Eisenblaetter M, Antoch G, Schönberg SO, Barkhausen J et al (2020) Work and Training Conditions of German Residents in Radiology—Results from a Nationwide Survey Conducted by the Young Radiology Forum in the German Roentgen Society. Rofo 192:458–470 31918440 10.1055/a-1047-1075

[3] Molwitz I, Eisenblätter M (2023) Digitale Tools zur Umsetzung strukturierter curriculumbasierter Weiterbildung in der Radiologie. Die Radiol 63:46–48 10.1007/s00117-022-01075-7 36178499

[4] Albrecht L, Maurer MH, Seithe T, Braun J, Gummert R, Auer J, Sponheuer K, Meyl TP, Hamm B, de Bucourt M (2018) Development of Report Turnaround Times in a University Department of Radiology during Implementation of a Reformed Curriculum for Undergraduate Medical Education. Rofo 190:259–264 28934807 10.1055/s-0043-118482

[5] Molwitz I, Kemper C, Stahlmann K, Oechtering TH, Sieren MM, Afat S, Gerwing M, Bucher AM, Storz C, Langenbach MC, Reim M, Lotz J et al (2023) Work expectations, their fulfillment, and exhaustion among radiologists of all career levels: what can be learned from the example of Germany. Eur Radiol 33:5664–5674 36897346 10.1007/s00330-023-09510-6 PMC9999063

[6] Rehani B, Zhang YC, Rehani MM, Palkó A, Lau L, Lette MN, Dillon WP (2017) Radiology education in Europe: Analysis of results from 22 European countries. World J Radiol 9:55–62 28298965 10.4329/wjr.v9.i2.55 PMC5334502

[7] Barkhausen J, Mentzel J, Zimmer C (2022) Strukturierung der Weiterbildung im Gebiet Radiologie an Zentren mit Schwerpunktabteilungen. Rofo 194:671–672 35613569 10.1055/a-1814-0119

[8] (2024) Gemeinsame Stellungnahme des Forums Junge Radiologie, der Jungen Kinderradiologie und Jungen Neuroradiologie. https://www.forum-junge-radiologie.de/de-DE/10037/stellungnahme-mbwo/. Zugegriffen: 29. Jan. 2025

[9] Sieren M, Katoh M, Mahnken AH, Reimer P, Westphalen K, Hoffmann RT, Paprottka P, Rohde S, Wacker FK, Minko P, Molwitz I, Oechtering TH et al (2022) Work and Training Conditions of German Residents and Young Radiologists in Interventional Radiology—A Nationwide Survey. Rofo 194:1346–1357 35830856 10.1055/a-1853-8549

